# Norovirus infection in pediatric acute gastroenteritis: epidemiology and clinical features in Ezhou, China, 2023–2025

**DOI:** 10.3389/fphys.2026.1808306

**Published:** 2026-08-05

**Authors:** Dan Luo, Siyue Tang, Yingru Liu, Shan Guo, Zhi Yu, Meichan Xu, Jiachan Zhang, Huifen Huang, Qiaoyu Pan, Lijuan Huang, Fei Wang, Yuan Gao

**Affiliations:** 1 Department of Gastroenterology, Wuhan Children’s Hospital, Tongji Medical College, Huazhong University of Science and Technology, Wuhan, China; 2 Graduate Training Base of Wuhan Children’s Hospital, Wuhan University of Science and Technology, Wuhan, China; 3 School of Medicine, Faculty of Medicine, Wuhan University of Science and Technology, Wuhan, China; 4 Department of Pediatrics, Ezhou Maternal and Child Health Hospital, Ezhou, Hubei, China; 5 Department of Clinical Laboratory, Ezhou Maternal and Child Health Hospital, Ezhou, Hubei, China

**Keywords:** norovirus (NoV), acute gastroenteritis (AGE), epidemiology, clinical characteristics, fecal samples, colloidal gold assay, sequencing analysis, pediatric patients

## Abstract

**Objective:**

Norovirus (NoV) is a major pathogen causing acute gastroenteritis (AGE) worldwide, imposing a substantial disease burden. This study aimed to investigate the epidemiological, clinical and genotypic characteristics of NoV infection in pediatric patients with AGE in Ezhou, central China.

**Methods:**

Between January 2023 and June 2025, we enrolled pediatric AGE patients from Ezhou Maternal and Child Health Hospital and collected their demographic profiles, clinical records and stool specimens, together with matched local meteorological data. All stool samples were screened for NoV using a commercial colloidal gold immunoassay kit. A randomly selected subset of NoV−positive samples was further subjected to genetic sequencing.

**Results:**

Among the 8,435 enrolled pediatric patients, 422 (5.0%) tested positive for NoV, with annual positivity rates of 4.24% (2023), 5.89% (2024), and 4.57% (January-June 2025). The positive cases comprised 259 males and 163 females. Children aged 1–2 years had the highest NoV positivity rate. The epidemic period spanned winter to spring, corresponding to ambient humidity of 66.5%–69.95%. Typical clinical presentations included fever, vomiting, watery diarrhea, dehydration, abdominal pain, convulsions, lethargy and electrolyte disturbances. Genetic sequencing identified three NoV genotypes across the 2.5-year surveillance period: GII.17, GII.3 and GII.4.

**Conclusions:**

NoV infection prevalence rose continuously in Ezhou during the 2.5-year study period, with seasonal peaks in winter and spring. GII.17 was the dominant genotype among sequenced isolates. Children aged 1–2 years constitute the highest-risk population and exhibit a wide range of clinical manifestations. Fecal colloidal gold immunoassay serves as a practical screening tool for NoV detection in pediatric populations at primary medical institutions.

## 1 Introduction

Acute gastroenteritis (AGE) remains a leading cause of morbidity and mortality in pediatric patients globally, with viral pathogens being the primary contributors (Lee et al., 2025; Sikenis et al., 2026). The predominant viral agents fall into two families: *Reoviridae* (represented by rotavirus) and *Caliciviridae* (including NoV and sapovirus) (Li N. et al., 2024). Following the widespread rollout of rotavirus vaccines, NoV has become the predominant pathogen triggering both large-scale global outbreaks and sporadic AGE cases (Carlson et al., 2024; Parra et al., 2025). It accounts for roughly 20% of all AGE episodes and causes more than 200,000 annual deaths in low- and middle-income nations (Sarmento et al., 2023). By comparison, sapovirus primarily induces isolated sporadic infections, with a prevalence rate of only 1.73%-2.2%—far lower than that of NoV (Lee et al., 2025; Sikenis et al., 2026).

NoV is a non-enveloped positive-sense single-stranded RNA virus with a ~7.5 kb genome containing three open reading frames (ORFs) (Sarmento et al., 2023; Capece and Tobin, 2026). ORF1 encodes non-structural proteins including RdRp, while ORF2 and ORF3 separately produce major capsid VP1 and minor capsid VP2 (Lee et al., 2022). Molecular surveillance reveals persistent recombinant strain emergence via altered RdRp-VP1 pairing, with recombination hotspots located at the ORF1-ORF2 boundary (Mathew et al., 2019). Classification based on VP1 amino acid variation defines ten genogroups (GI-GX) with 48 genotypes, yet merely five (GI, GII, GIV, GVIII, GIX) infect humans (Chhabra et al., 2020; John et al., 2021). GI and GII are the primary pathogenic genogroups; GII.4, the dominant genotype, has caused most global NoV outbreaks in the past two decades (van Beek et al., 2018; Gentile et al., 2023). Pandemic GII.4 variants have emerged every 3–5 years since the mid-1990s, triggering recurring cross-continental epidemics (Parra, 2019). Successive strain replacement occurs when novel variants accumulate VP1 epitope (A-I) mutations to evade herd immunity and gain transmission superiority (Mallory et al., 2019). Some GII.4 variants circulate within limited regions, such as the Hong Kong GII.4 strain widely detected across Asian and European surveillance platforms from 2017 to 2019 (Chan et al., 2021).

Global surveillance of NoV faces considerable obstacles that complicate accurate burden estimation. Data on NoV prevalence and genotype circulation remain sparse and substantially underreported, particularly in developing countries. Recent findings have revealed marked epidemiological heterogeneity and extensive genetic diversity of NoV worldwide (Hernandez et al., 2018; Fang et al., 2021). In China, previous studies report a high NoV incidence (26.4%-27.6%) among acute gastroenteritis cases, alongside notable genetic variability over the same period (Fang et al., 2021; Fang et al., 2022).

In Central China, the potential shifts in NoV epidemiological patterns—including seasonal distribution, age-stratified disease burden, and circulating genotypes—and their overall population impact remain poorly characterized. This study represents the first systematic report describing the epidemiological, clinical, and genetic features of NoV infections in Ezhou, Central China.

## 2 Materials and methods

### 2.1 Study population and clinical data collection

This study was conducted from January 2023 to June 2025 and enrolled 8,435 pediatric inpatients from Ezhou Maternal and Child Health Hospital. Inclusion criteria were: (1) age < 18 years, (2) a clinical diagnosis of AGE, and (3) suspected NoV infection based on clinical presentation. The study was approved by the Ethics Committee of Ezhou Maternal and Child Health Hospital. Written informed consent was obtained from the parents or legal guardians of all participants, and patient data were anonymized and securely stored. Epidemiological, clinical, and laboratory data were extracted from electronic medical records.

### 2.2 Sample collection

This study included stool samples collected from pediatric inpatients with AGE. AGE was clinically defined as the passage of three or more loose or watery stools within a 24−hour period. Stool specimens and clinical records were obtained from children attending the Department of Pediatrics at Ezhou Maternal and Child Health Hospital. Samples were stored at 4 °C in the hospital’s clinical laboratory and underwent centralized testing each afternoon.

### 2.3 NoV detection by colloidal gold assay

Detection was performed according to the manufacturer’s instructions using a commercially available, China−licensed NoV antigen detection kit (colloidal gold method; Beijing Xinxingsihuan Biotechnology Co., Ltd.), specifically designed for qualitative detection of the common NoV antigen (shared by GI and GII) in human stool specimens. In internal validation, this kit showed 100% sensitivity and specificity against reference panels. Each sample was tested in triplicate independently by different researchers, and only concordant results were considered valid.

### 2.4 NoV RNA extraction and sequencing

Ten stool samples positive for NoV by colloidal gold assay were randomly selected for sequencing. Each sample was prepared as a 10% (w/v) suspension in phosphate−buffered saline, clarified by centrifugation, and then subjected to RNA extraction using TRNzol Universal Reagent according to the manufacturer’s protocol. Sequencing libraries were constructed with the Fast RNA−seq Lib Prep Kit V2, quality−checked, pooled at equimolar ratios, and sequenced on a BGI platform with 2×150 bp paired−end reads. Finally, seven complete sequences were obtained and deposited in GenBank under accession numbers PZ580601- PZ580606 (one additional sequence is currently pending submission).

### 2.5 Phylogenetic analysis of NoV genomes

Nucleotide sequences were aligned in MAFFT, and phylogenetic relationships among NoV strains were inferred using Kimura’s substitution model for DNA and protein sequences. Phylogenetic trees based on the capsid and RdRp gene sequences were constructed via the neighbor-joining algorithm in FastTree, with bootstrap validation performed using 1000 replicates.

### 2.6 Statistical analysis

All statistical analyses were conducted with SPSS Statistics version 19.0. Categorical variables were compared via the chi-square test, while continuous variables were analyzed using Student’s t-test. A P value less than 0.05 was defined as the threshold for statistical significance.

## 3 Results

### 3.1 The epidemiological characteristics of NoV infection in pediatric patients with AGE

**Figure 1:**
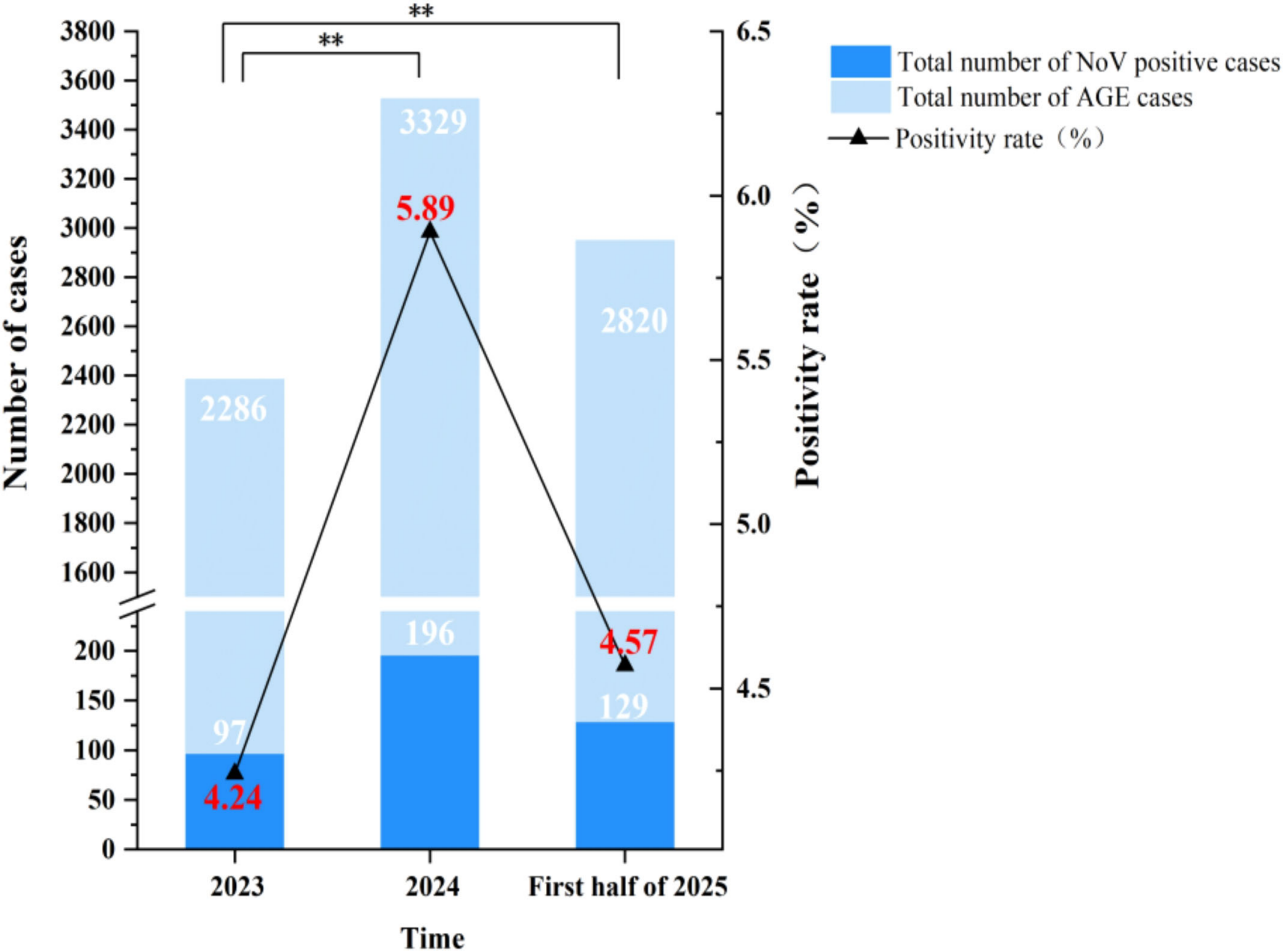
NoV detection rates in pediatric AGE patients from 2023 to first half of 2025. Over 2.5 years, among 8,435 stool samples from pediatric AGE patients, 5.0% (422) were NoV-positive, with significantly lower rates in 2023 (4.24%) than in 2024 (5.89%) and first half of 2025 (4.57%) (*P<0.01*). (**denotes *P<0.01*. The axis break on the vertical axis is used to optimize the overall data visualization).

Over this two-and-a-half-year study period, stool specimens collected from 8,435 hospitalized children presenting with AGE were screened for NoV. Overall, 422 specimens yielded NoV-positive results, corresponding to an overall positivity rate of 5.0%. Stratified by time frame, the positivity rates stood at 4.24% (97/2,286) in 2023, 5.89% (196/3,329) in 2024, and 4.57% (129/2,820) in the first six months of 2025 (**Figure 1**). Statistical comparison demonstrated that the NoV positivity rate in 2023 was markedly lower than those recorded in 2024 and the first half of 2025 (P < 0.01). When calculating the proportional composition of all detected NoV-positive cases, the distribution across time periods was 22.99% (97/422) for 2023, 46.44% (196/422) for 2024, and 30.57% (129/422) for the first half of 2025.

**Figure 2:**
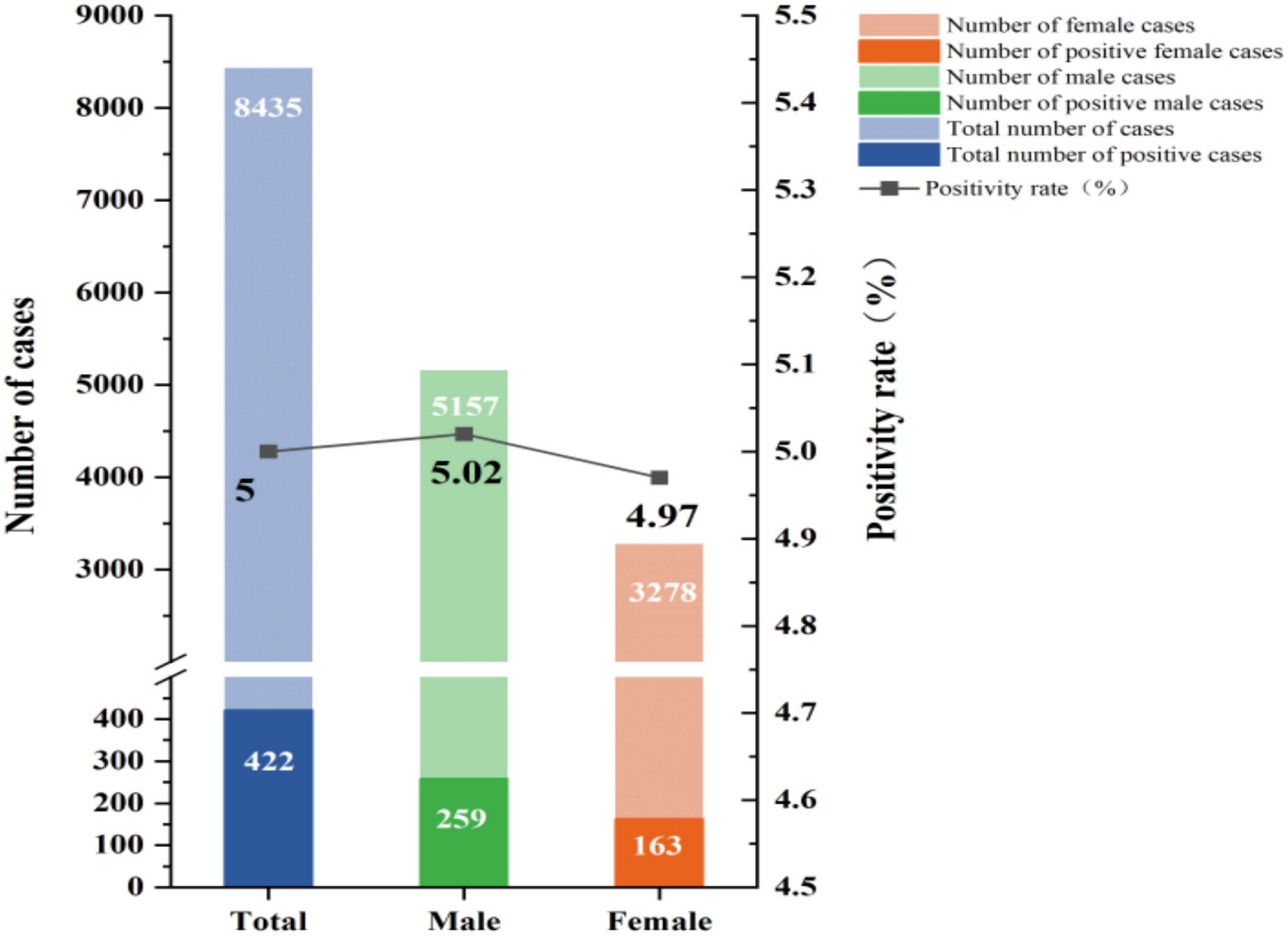
Sex distribution of enrolled pediatric AGE patients and NoV-positive cases. Among 422 NoV-positive cases, 61.37% (259) were male, 38.63%(163)were female, with no significant gender difference.

Of the 8,435 pediatric patients enrolled with AGE, 5,157 (61.14%) were male and 3,278 (38.86%) were female. Among the 422 NoV-positive individuals, 259 (61.37%) were male and 163 (38.63%) were female, and no statistically significant intergroup difference was observed by sex (**Figure 2**).

**Figure 3:**
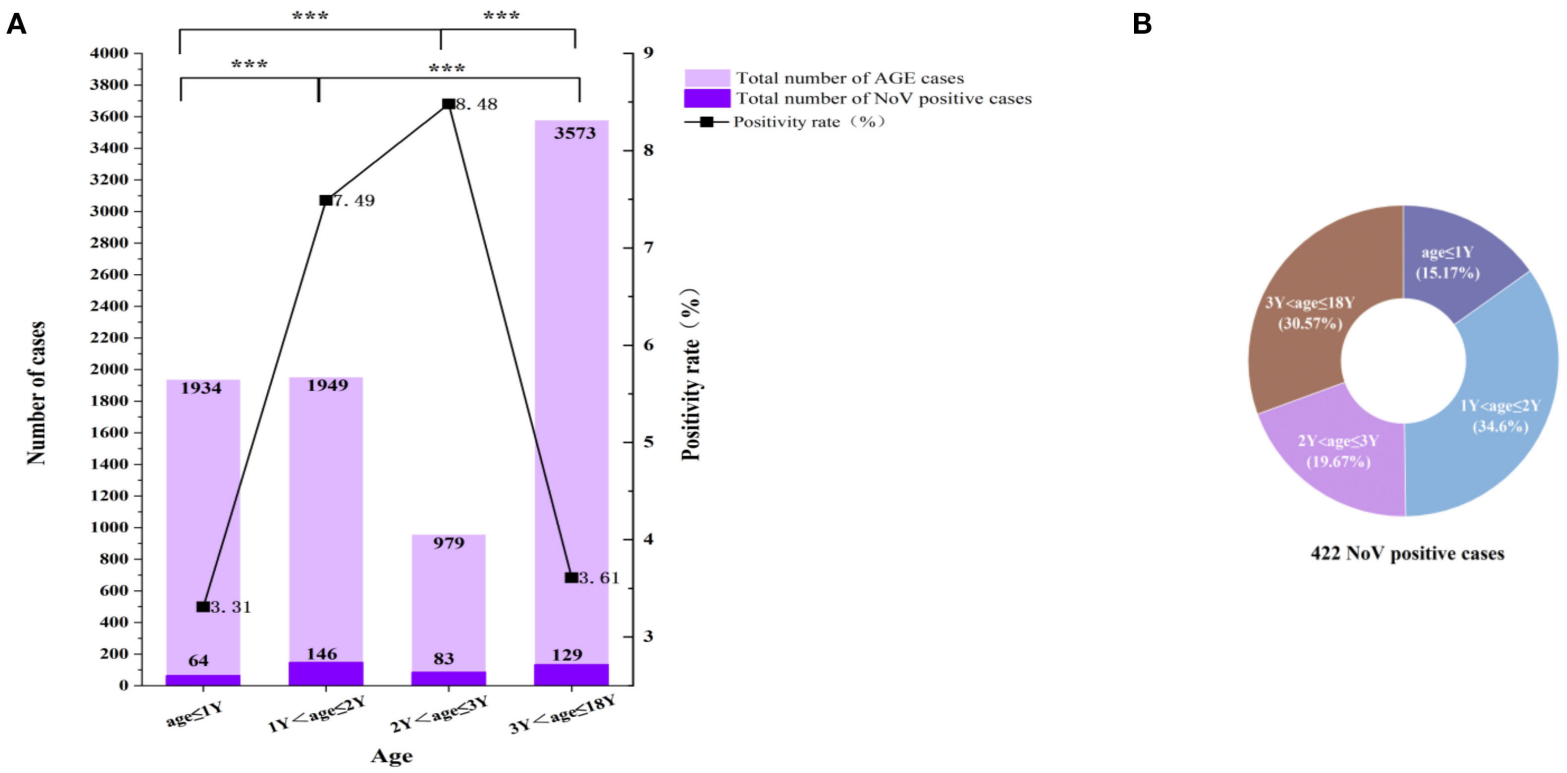
Age−stratified NoV detection rates and the percentage distribution of NoV−positive cases by age group among pediatric patients with AGE. ** denotes P < 0.001 **(A)** Among pediatric AGE patients, NoV detection rates were significantly higher in the 1–2 years (7.49%) and 2–3 years (8.48%) groups than in ≤1 year (3.31%) and 3–18 years (3.61%) (***P<0.001). **(B)** Among the 422 positive cases, the age group distribution was as follows: 15.17% (≤1 year), 34.6% (1–2 years), 19.67% (2–3 years), and 30.57% (3–18 years).

AGE was diagnosed in children spanning all age strata. Patient counts stratified by age (≤ 1, 1-2, 2-3, and 3–18 years) were 1,934, 1,949, 979, and 3,573, respectively. Norovirus was detected in every age subgroup. The positivity rates were significantly higher in children aged 1–2 years (7.49%, 146/1,949) and 2–3 years (8.48%, 83/979) than in those aged ≤ 1 year (3.31%, 64/1,934) and 3–18 years (3.61%, 129/3,573) (*P<0.001*) (**Figure 3A**). When stratified by age, the proportional composition of all 422 NoV-positive cases was 15.17% (64/422), 34.60% (146/422), 19.67% (83/422), and 30.57% (129/422) (**Figure 3B**).

**Figure 4:**
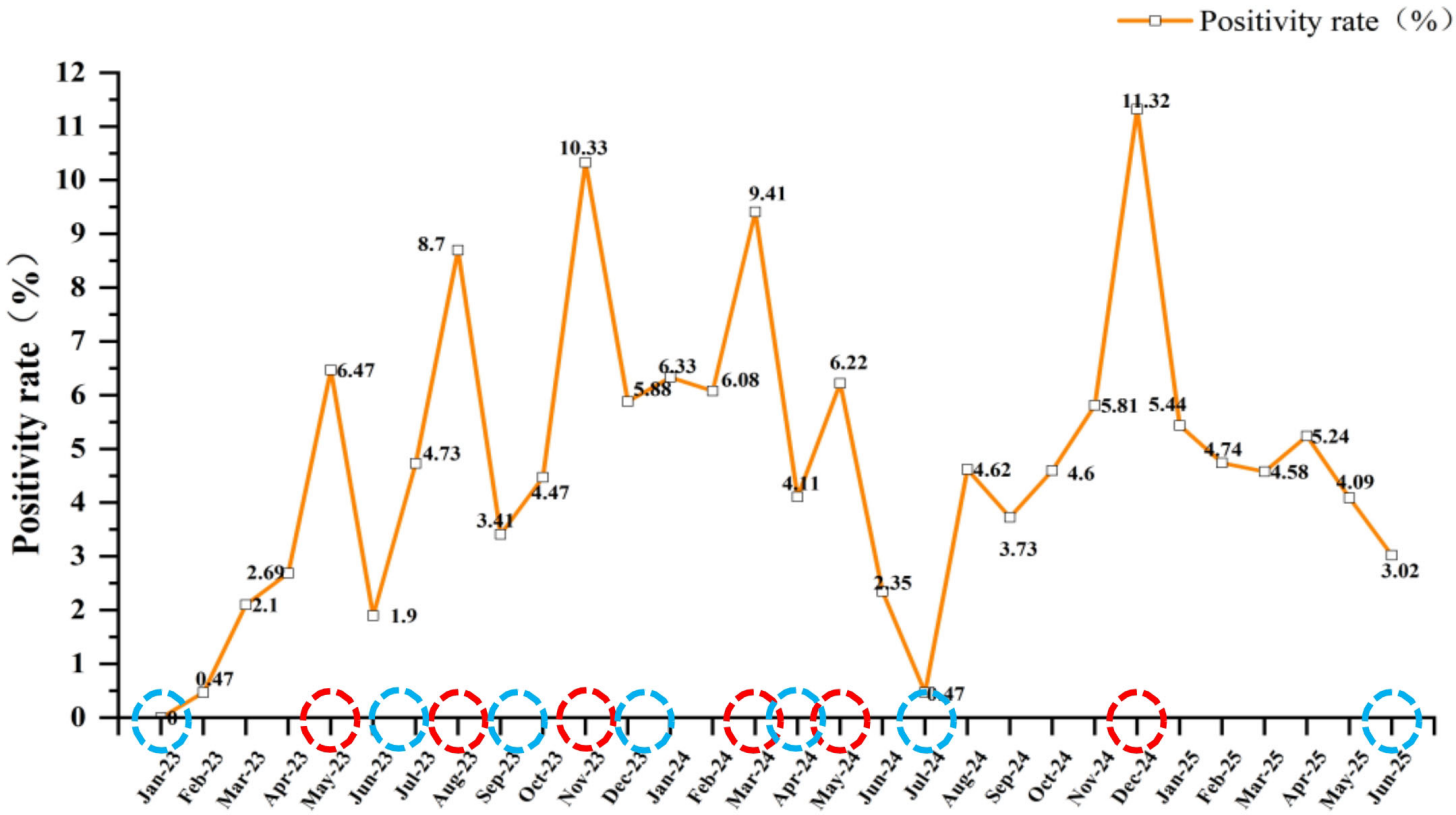
Monthly NoV detection rates. During the 2.5-year study period, monthly NoV detection rates showed six peaks: May 2023 (6.47%), August 2023 (8.7%), November 2023 (10.33%), March 2024 (9.41%), May 2024 (6.22%), and December 2024 (11.32%). They also showed seven troughs: January 2023 (0%), June 2023 (1.9%), September 2023 (3.41%), December 2023 (5.88%), April 2024(4.11%), July 2024 (0.47%), and June 2025 (3.02%).

Over the 2.5−year study period that concluded in June 2025, monthly NoV detection rates showed substantial fluctuations. Six activity peaks were observed: May, August, and November 2023 (6.47%, 8.70%, and 10.33%, respectively), as well as March, May, and December 2024 (9.41%, 6.22%, and 11.32%, respectively). Correspondingly, seven troughs were identified: January (0.00%), June (1.90%), September (3.41%), and December (5.88%) in 2023; April (4.11%) and July (0.47%) in 2024; and June (3.02%) in 2025 (**Figure 4**).

**Figure 5:**
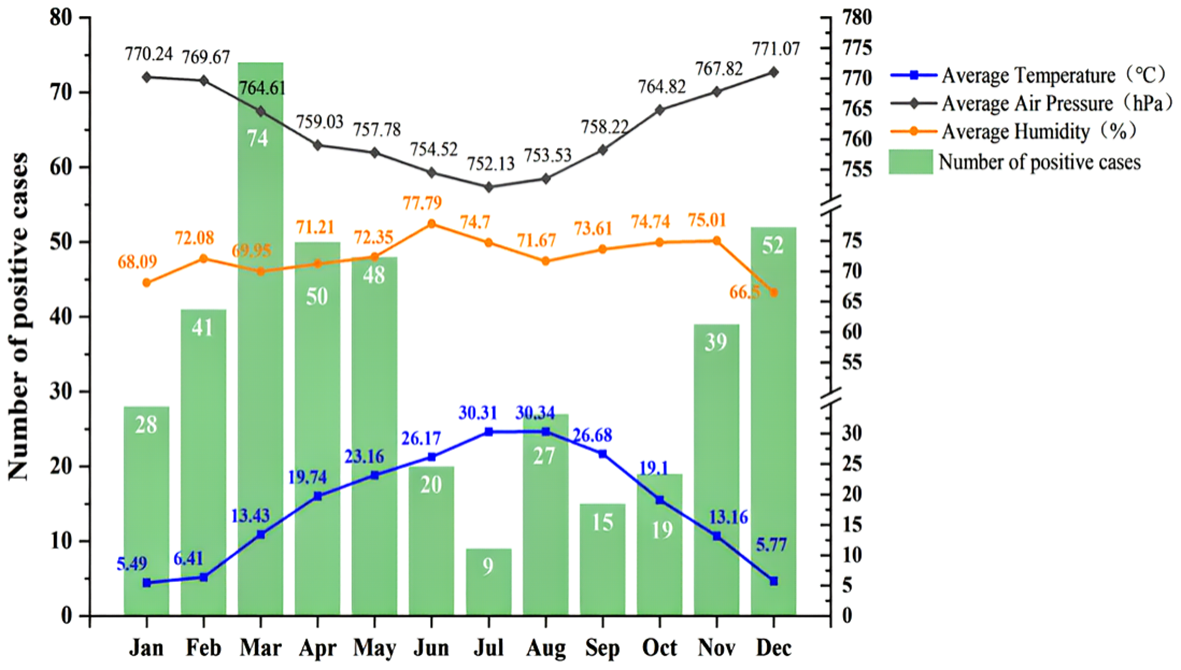
Association of temperature and atmospheric pressure with NoV-positive cases in pediatric AGE patients. NoV-positive cases in pediatric AGE patients were higher when average temperatures were lower (5.49–23.16 °C) and atmospheric pressure was higher (757.78–771.07 hPa), and lower during hot months with lower pressure.

Subsequently, this study further explored correlations between climatic variables and NoV infection rates. For temperature, NoV-positive counts rose at mean temperatures of 5.49-23.16 °C (January-May), fell markedly near 30 °C (June-October), and rebounded at roughly 13.16 °C (November-December). Atmospheric pressure displayed an analogous seasonal trend: high case loads coincided with pressures of 757.78-770.24 hPa (January-May), low counts occurred under pressures below 754.52 hPa (June-October), and case numbers climbed again once pressure exceeded 767.82 hPa (November-December) (**Figure 5**).

**Figure 6:**
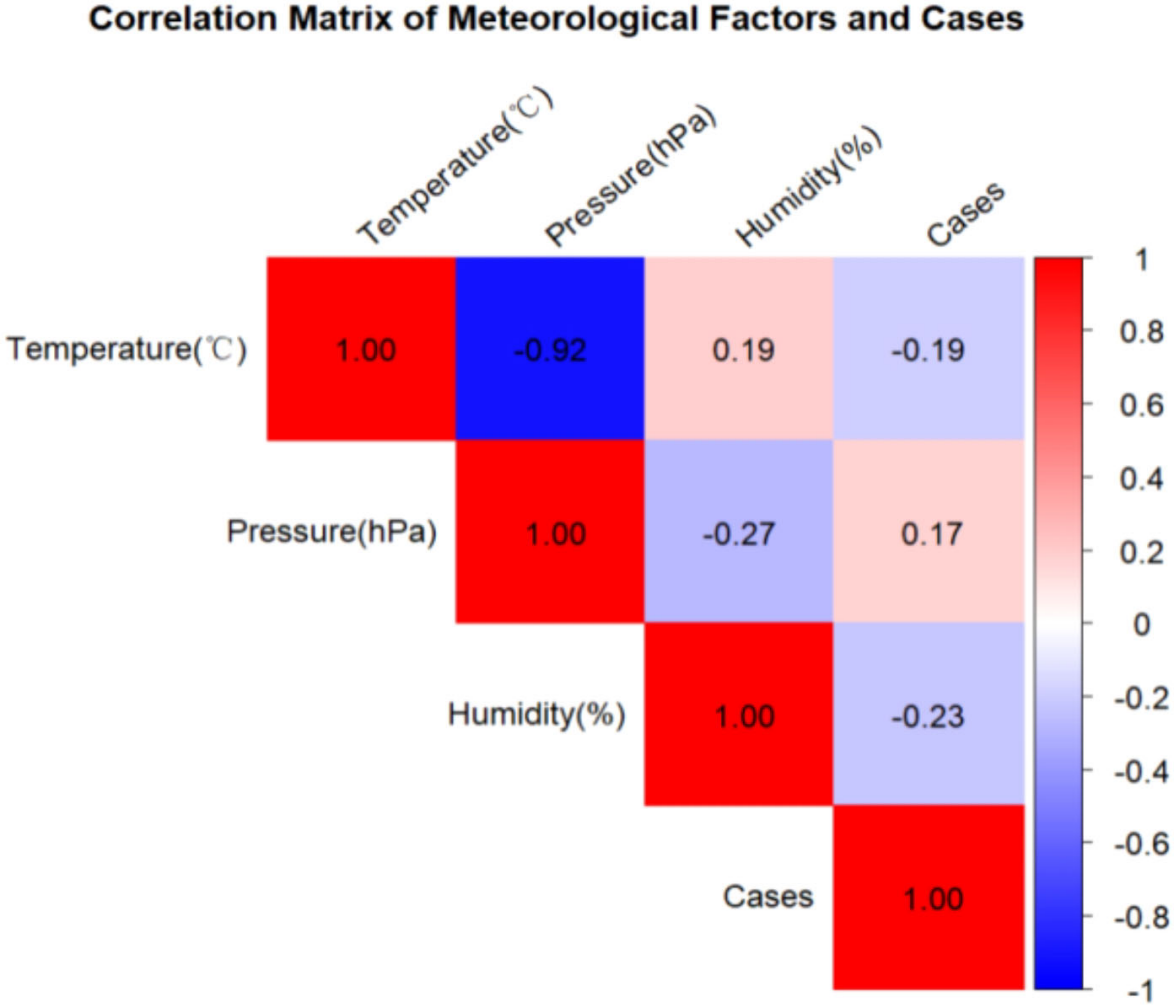
Correlations of climatic factors (temperature, pressure, humidity) with NoV case numbers. Temperature showed a very weak negative correlation, atmospheric pressure a very weak positive correlation, and humidity a weak negative correlation with case numbers.

Further analyses demonstrated a very weak negative correlation between temperature and case counts, alongside a very weak positive correlation for atmospheric pressure. In comparison, humidity was weakly negatively correlated with NoV case numbers (**Figure 6**).

### 3.2 Clinical features of NoV infection in pediatric patients with AGE

**Figure 7:**
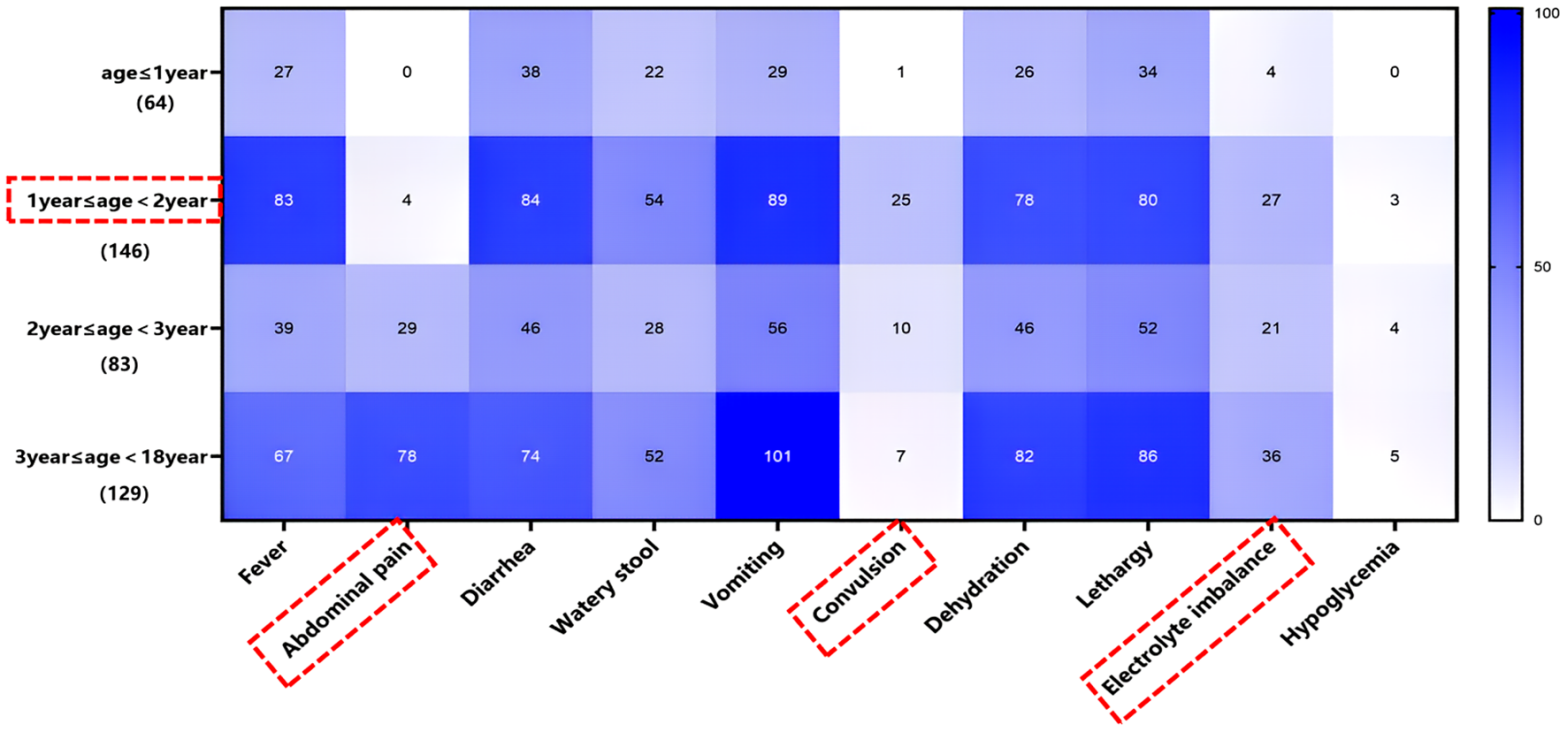
Clinical characteristics of 422 children with AGE caused by NoV. Symptom profile and age-stratified manifestations of NoV infection in pediatric patients. Among 422 pediatric NoV patients, the most common symptoms were vomiting (65.2%), lethargy (59.7%), diarrhea (57.2%), dehydration (55.0%), and fever (51.2%), with age-specific patterns varying by group; convulsions were concentrated in the 1–2 years group (58.1%), abdominal pain in the 3–18 years group (70.3%), and hypoglycemia was rare (2.8%).

Symptoms among pediatric patients with NoV infection included fever, abdominal pain, diarrhea, vomiting, convulsions, dehydration, lethargy, electrolyte imbalances, and hypoglycemia. Among the 422 infected patients, 51.18% (216/422) presented with fever, 26.30% (111/422) with abdominal pain, 57.25% (242/422) with diarrhea, 65.17% (275/422) with vomiting, 54.98% (232/422) with dehydration, 10.19% (43/422) with convulsions, 59.72% (252/422) with lethargy, 20.85% (88/422) with electrolyte imbalances, and 2.84% (12/422) with hypoglycemia (**Figure 7**).

Age−stratified analysis revealed that the top three symptoms were diarrhea, lethargy, and vomiting (in that order) in the ≤1-year-old group; vomiting, diarrhea, and fever in the 1-2-year-old group; vomiting, lethargy, and diarrhea in the 2-3-year-old group; and vomiting, lethargy, and dehydration in the 3-18-year-old group (**Figure 7**).

Additionally, convulsive cases in the 1-2-year-old group accounted for 58.14% (25/43) of all patients with convulsions, whereas abdominal pain cases in the 3-18-year-old group represented 70.27% (78/111) of all patients with abdominal pain. Electrolyte imbalances were mainly observed in patients older than 1 year. Hypoglycemia was rare, with proportions of 0.00%, 2.10%, 4.80%, and 3.88% across the four age groups, respectively (**Figure 7**).

### 3.3 Genotypic composition and phylogenetic analysis of NoV in clinical samples

**Figure 8:**
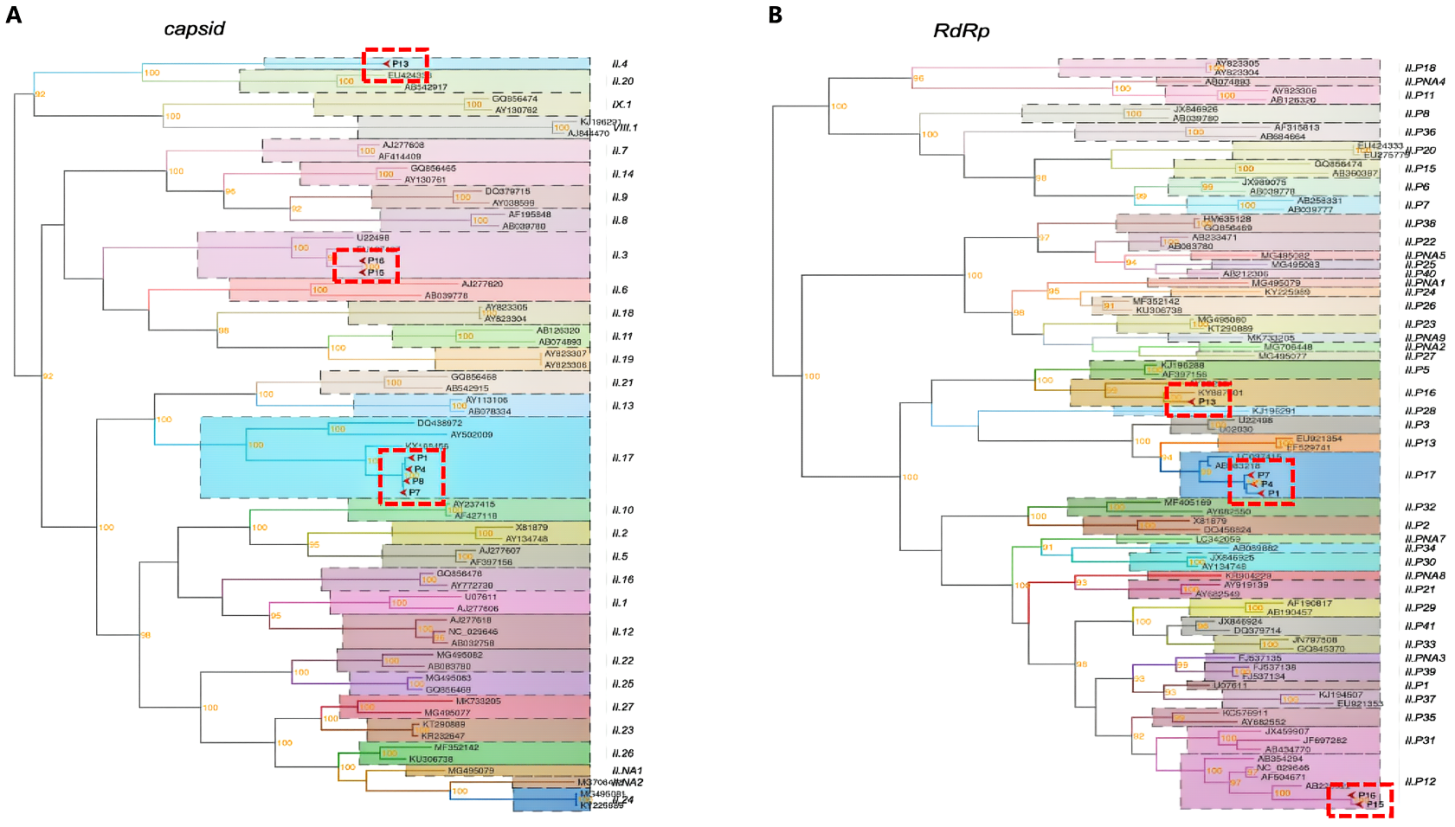
Genotypic characterization of the 10 sequenced NoV strains revealed that GII.17 was the dominant genotype, accounting for 4 of 7 capsid gene sequences **(A)** and 3 of 6 polymerase gene sequences **(B)**.

Ten stool specimens were randomly selected for NoV genetic sequencing. Full capsid protein gene sequences were successfully recovered from seven strains: one GII.4, two GII.3, and four GII.17 (**Figure 8A**). Meanwhile, complete RNA polymerase gene sequences were obtained from six strains: one GII.P16, two GII.P12, and three GII.P17 (**Figure 8B**). These results suggest that GII.17 constituted the dominant genotype among sequenced isolates.

## 4 Discussion

Viral AGE remains a significant cause of morbidity and mortality in developing countries (Kachooei et al., 2025). Notably, as RV vaccination has been widely implemented in some countries and reduced RV-associated diarrhea, NoV has now replaced rotavirus as the leading cause of pediatric AGE (Capece and Tobin, 2025; Prasad et al., 2025). While most previous studies have focused on provincial capitals or metropolitan areas in higher-income settings, the prevalence of NoV in pediatric patients from smaller cities or rural areas in lower-income settings deserves more attention (Chigor et al., 2024). To address this gap, the present study investigated the epidemiological, clinical, and molecular characteristics of NoV infection in pediatric AGE patients in Ezhou City between 2023 and the first half of 2025, aiming to provide an objective basis for optimizing prevention and control strategies and alleviating the disease burden.

This study revealed that the NoV positivity rate among pediatric AGE patients rose from 4.24% in 2023 to 5.89% in 2024 and stayed elevated at 4.57% in the first half of 2025, with the 2024 and mid−2025 rates significantly higher than the 2023 baseline. By comparison, NoV detection rates reported over comparable periods were 19.60% in Beijing (Ai et al., 2025), 14.64% in Hubei (2017-2019) (Li et al., 2023), 1.50% across Northern and Northwestern China (2016-2020, including 28,189 hospitalized children from 27 hospitals) (Li F. et al., 2024), 13.70% in Palermo, Italy (2003-2013, among 4,725 hospitalized children) (Amodio et al., 2022), and 11.93% (73/612) in Yunnan (2020-2022, pediatric AGE patients aged <5 years) (Li N. et al., 2024). Such marked spatial and temporal disparities highlight the necessity of continuous, targeted surveillance and intervention strategies. In absolute case counts, 97 NoV-positive patients were identified in 2023; this figure doubled in 2024, and the number recorded within only the first half of 2025 was already 1.33 times the full−year case count for 2023. Accordingly, a distinct year−on−year rising trend was observed across the three study years. This rebound aligns with findings from Hangzhou, where NoV-positive cases in 2023 accounted for 63.10% of all specimens collected throughout 2021-2023 (Chen D. et al., 2024). Likewise, a dramatic upswing in NoV-linked AGE during the 2023–2024 season has been documented in the United States, Austria, Germany, Ireland, the Netherlands, and England (Chhabra et al., 2024; Capece and Tobin, 2025).

In this study, a total of 422 NoV−positive cases were identified (259 boys and 163 girls), with no significant between-group difference in detection rates. This result aligns with previous investigations conducted in Tianjin, Hangzhou, and southwestern China (Fang et al., 2022; Yang et al., 2022; Chen D. et al., 2024). Nevertheless, research from Qatar and Harbin has documented higher NoV infection rates among boys compared with girls (Mathew et al., 2019; Tian et al., 2025). Those studies revealed marked sex−related disparities in pediatric NoV infection, indicating that gender could serve as a potential susceptibility modifier. Such inconsistencies are probably attributable to regional discrepancies in the gender composition of study populations and divergent lifestyle behaviors.

In this study, 422 NoV-positive cases were categorized into four age groups: <1 year, 1–2 years, 2–3 years, and 3–18 years. The broad 3–18 years range was treated as a single group because the case distribution within it was highly uneven, which facilitated statistical analysis. The positivity rates for the four groups were 3.31%, 7.49%, 8.48%, and 3.61%, respectively, with the 1-2−year-old and 2-3−year-old groups showing significant differences from the other two groups. In terms of absolute case numbers, the 1-2−year-old group had the highest count. A study conducted six years ago in Hubei Province reported the highest detection rate (19.76%) in pediatric patients aged 7–12 months (Li et al., 2023), further confirming that infants and young children are a high−risk population. Although the 3-18−year-old group ranked second in total case numbers, its numbers per specific age were relatively low — far fewer than the 64 cases in the <1 year group and the 83 cases in the 2-3-year group. Notably, patients under 3 years of age accounted for 69.43% of all 422 positive cases, underscoring children under 3 years as a key target population. Previous studies have also reported a higher risk among preschool children, with those aged 0–5 years constituting the majority of suspected and confirmed NoV-associated AGE cases (Mathew et al., 2019; Tian et al., 2025). Therefore, enhanced protection for infants and young children could substantially reduce pediatric NoV infection rates.

Although NoV infection can occur year−round, monthly detection rates displayed marked temporal fluctuations across the study timeframe. Over the 2.5-year observation period, six epidemic peaks were detected: March, August, and November 2023, as well as March, May, and December 2024. Correspondingly, seven troughs were recorded: January, June, September, and December 2023; April and July 2024; and June 2025. Stratified by season (spring: March-May; summer: June-August; autumn: September-November; winter: December-February), 50.00% of all peaks fell in spring (three peaks), 16.70% in summer (one peak), and 33.30% in winter (two peaks). In contrast, troughs accounted for 14.30% in spring (one trough), 42.90% in summer (three troughs), 14.30% in autumn (one trough), and 28.60% in winter (two troughs). Therefore, epidemic peaks predominantly clustered in spring and winter (83.30%), while low-incidence troughs were most common in summer and autumn (57.10%). This seasonal distribution is largely consistent with previous studies reporting predominant NoV circulation during cooler months (Kreidieh et al., 2017; Kambhampati et al., 2023; Tomb et al., 2024; Xu et al., 2024). Of note, a single summer peak emerged in August — consistent with epidemiological findings from Greece and Libya — though Ezhou has a subtropical monsoon climate, while those two regions feature Mediterranean climates. Despite such climatic disparities, multiple determinants including dietary customs, human mobility, and local public health infrastructure jointly regulate local NoV prevalence (Abugalia et al., 2011; Tryfinopoulou et al., 2019). Accordingly, clinicians and guardians should sustain intensified surveillance for pediatric AGE throughout epidemic and inter−epidemic intervals alike.

Given the clear seasonal pattern of NoV infections, we further explored the association between climatic factors and case numbers. Temperature exhibited a clear inverse relationship with cases: lower temperatures in early years corresponded to higher case numbers, while warming periods saw declines, and cooling periods brought rebounds. By contrast, atmospheric pressure showed a similar directional trend as cases, while humidity varied only weakly with case fluctuations. Correlation analyses among temperature, pressure, humidity, and case numbers revealed that pressure was very weakly positively correlated with cases, temperature was very weakly negatively correlated, and humidity was weakly negatively correlated. Notably, humidity had a stronger negative association than both temperature and pressure — lower humidity tended to coincide with higher NoV case counts.

A study from Roraima, the Amazon region of Brazil involving 941 children showed that relative humidity had the strongest effect on NoV positivity rates, in drier periods there was an increase in the NoV detection rate. This was consistent with our research findings. A study from Qatar involving 177 children showed that the number of NoV infections decreased from October to December (winter, with an average temperature of 22 °C and average humidity of 66%), while an upward trend was observed during the warmer months of May to August (average temperature 37 °C, average humidity 48%). This study also indicated that lower humidity is associated with a higher number of cases, and furthermore, that humidity has a greater influence on case numbers than temperature. This was also in line with our findings. Ezhou is in central China, in the eastern part of Hubei Province, within the middle and lower reaches of the Yangtze River. It experiences a subtropical monsoon climate with moderate with low rainfall in winter and early spring, characterized by low temperatures and low humidity. Consequently, strengthening NoV prevention measures during these two seasons is particularly important for pediatric patients in the Ezhou region.

In this study, the clinical manifestations observed in pediatric patients with NoV infection included fever, abdominal pain, diarrhea, vomiting, dehydration, electrolyte imbalances, lethargy, convulsions, and hypoglycemia. Literature also reported that common symptoms of NoV infection were nausea, vomiting, and diarrhea, with vomiting often being the initial symptom and potentially lasting 1–3 days (Chen J. et al., 2024; Capece and Tobin, 2025). In this study, patients aged 1–2 years and 3–18 years accounted for a relatively large proportion of the cases, and the frequency of the aforementioned symptoms was also higher in these groups. It is worth noting that children aged 1–2 years had the highest cases of convulsions across all age groups and no one developed epilepsy. Therefore, close monitoring of neurological symptoms should be emphasized for patients in this age range. This finding is consistent with reports from other researchers (Shi et al., 2023). Electrolyte imbalances were more frequently distributed among patients older than 1 year, underscoring the need for enhanced intervention and management of clinical symptoms in these cases. The frequency of abdominal pain increased with age, which might be related to improved ability to express subjective discomfort. The literature also reported that infections with non-GII.4 genotypes were more likely to present with abdominal pain and dehydration (Fang et al., 2022).

NoV possesses remarkable genetic diversity, with numerous genogroups and genotypes cocirculating worldwide and triggering human gastroenteritis (Carlson et al., 2024). Prevalent epidemic genotypes comprise GII.4, GII.3, GII.2, GII.17, and GII.6, and GII.4 has long been recognized as the globally dominant genotype (Zhou et al., 2020; Lu et al., 2022; Chaimongkol et al., 2025). By contrast, random genetic sequencing performed in the present study identified GII.17 as the prevailing isolate among sequenced specimens, despite incomplete capsid sequences from three isolates and partial RNA-dependent RNA polymerase (RdRp) sequences from four isolates. This genotype shift aligns with international surveillance data describing surging GII.17 outbreaks in Austria, Germany, France, Ireland, the Netherlands, England, and the United States throughout the 2023–2024 season, accompanied by a synchronous drop in GII.4 circulation; GII.17 accounted for 17%-64% of all GII NoV detections (Chhabra et al., 2024). Comparably, a Beijing-based epidemiological survey found GII.3 to be the leading genotype in 2023 (39.15%), followed by GII.4 (47.66%); in 2024, however, GII.17 gained predominance (41.43%), surpassing GII.4 (34.29%) and GII.3 (20.00%) (Ai et al., 2025). Taken together, these results demonstrate continuous dynamic turnover of dominant circulating NoV strains, underscoring the urgent requirement for persistent cross-regional NoV molecular surveillance.

This study has several limitations. First, only hospitalized children were recruited, and outpatient subjects were excluded; this design may underestimate the true NoV prevalence within Ezhou. Second, the colloidal gold assay is prone to false-negative and false-positive readings, which could introduce analytical bias. Third, constrained by limited funding, we obtained a modest number of sequencing specimens, and this small sample may fail to fully capture the full spectrum of NoV genotypes circulating locally. Fourth, only NoV testing was performed, with no parallel screening for other enteric viruses (e.g., rotavirus, astrovirus) or bacterial pathogens; therefore, mixed co-infections cannot be ruled out.

In summary, across the 2.5-year study period, NoV infection rates among pediatric patients in Ezhou rose year on year, with no statistically significant sex−related disparities. Children aged 1–3 years bore the highest infection burden, and winter and spring represented the epidemic peak seasons, which highlights the necessity of enhanced preventive interventions. Relative humidity exhibited a weak negative correlation with case counts. Typical clinical manifestations — fever, vomiting, diarrhea, dehydration, convulsions, and electrolyte imbalances — emphasized the value of close clinical observation and prompt symptomatic management. Predominant circulating NoV genotypes underwent dynamic shifts over the study timeframe, further stressing the demand for continuous cross−regional molecular surveillance. For small cities and rural settings such as Ezhou, the colloidal gold assay functioned as a convenient, reliable method for routine NoV screening.

## Data Availability

The datasets presented in this study can be found in online repositories. The names of the repository/repositories and accession number(s) can be found in the article/supplementary material.
